# Biomarkers for Heart Failure Prediction and Prevention

**DOI:** 10.3390/jcdd10120488

**Published:** 2023-12-06

**Authors:** Prasanti Alekhya Kotta, Vijay Nambi, Biykem Bozkurt

**Affiliations:** 1 Department of Internal Medicine, Baylor College of Medicine, Houston, TX 77030, USA; 2 Section of Cardiology, Department of Medicine, Baylor College of Medicine, Houston, TX 77030, USA; vnambi@bcm.edu; 3 Section of Cardiology, Michael E. DeBakey Veterans Affairs Medical Center, Houston, TX 77030, USA; 4 Department of Medicine, Cardiology Section, Winters Center for Heart Failure Research, Cardiovascular Research Institute, Baylor College of Medicine, DeBakey Veterans Affairs Medical Center, Houston, TX 77030, USA; bbozkurt@bcm.edu

**Keywords:** heart failure, biomarkers, screening, prediction, prevention, Stage B heart failure, pre-heart failure

## Abstract

Heart failure (HF) is a global pandemic affecting over 64 million people worldwide. Its prevalence is on an upward trajectory, with associated increasing healthcare expenditure. Organizations including the American College of Cardiology (ACC) and the American Heart Association (AHA) have identified HF prevention as an important focus. Recently, the ACC/AHA/Heart Failure Society of America (HFSA) Guidelines on heart failure were updated with a new Class IIa, Level of Evidence B recommendation for biomarker-based screening in patients at risk of developing heart failure. In this review, we evaluate the studies that have assessed the various roles and contributions of biomarkers in the prediction and prevention of heart failure. We examined studies that have utilized biomarkers to detect cardiac dysfunction or abnormality for HF risk prediction and screening before patients develop clinical signs and symptoms of HF. We also included studies with biomarkers on prognostication and risk prediction over and above existing HF risk prediction models and studies that address the utility of changes in biomarkers over time for HF risk. We discuss studies of biomarkers to guide management and assess the efficacy of prevention strategies and multi-biomarker and multimodality approaches to improve risk prediction.

## 1. Introduction 

Heart failure (HF) is a global pandemic affecting over 64 million people worldwide [1]. In the United States alone, about 6.7 million people are affected by HF. The lifetime risk of HF has increased to 24%, and the prevalence of HF is projected to exceed 8 million by 2030, with the associated healthcare expenditure projected to rise to USD 69.7 billion [2,3]. HF is associated with a poor prognosis with 50% survival at 5 years [4]. 

Organizations including the American College of Cardiology (ACC) and the American Heart Association (AHA) have identified HF prevention as an important focus. Recently, the ACC/AHA/Heart Failure Society of America (HFSA) Guidelines on heart failure were updated [5]. The previous 2013 ACC/AHA guidelines classified HF into stages A–D. Stage A included those at a high risk of HF development with the presence of risk factors such as hypertension, ischemic heart disease, obesity, and diabetes; Stage B included those with structural heart disease but without signs or symptoms of HF; Stage C included those with structural heart disease and prior or current symptoms of HF; and Stage D included those with refractory HF requiring advanced interventions. The 2022 guideline brought an important modification to this HF classification system with Stage B HF, which is now modified as pre-heart failure and includes elevated cardiac biomarkers as well as structural and functional cardiac abnormalities. Specifically, the guidelines recommend that individuals with elevated B-type natriuretic peptide (BNP), N-terminal pro-B-type natriuretic peptide (NT-proBNP), or persistently elevated levels of high-sensitivity cardiac troponins in the setting of exposure to cardiotoxic agents be classified as Stage B HF or pre-HF. The 2022 guidelines also provide a Class IIa, Level of evidence B recommendation for BNP or NT-proBNP-based screening for patients at risk of developing heart failure followed by team-based care, including a cardiovascular specialist optimizing goal-directed medical therapy (GDMT), as a strategy to prevent the development of new-onset HF and left ventricular dysfunction. 

Heart failure prevention entails two fundamental components. One, the ability to identify patients at risk, at an early preclinical stage of illness, and two, effective interventions that can prevent the development of the disease and its course. However, with HF being a heterogeneous, multifactorial disease involving complex pathophysiological processes and different risk factors, standardized screening and prevention of HF can be challenging. Regarding the first step, there is a large volume of growing literature on HF risk prediction scores and the use of biomarkers to identify those at increased risk of HF development [6]. Regarding the second step, several studies have addressed feasible strategies to alter HF disease progression. Both of these are discussed in the following sections. 

## 2. Biomarkers for the Prediction and Prevention of HF

**Figure 1:**
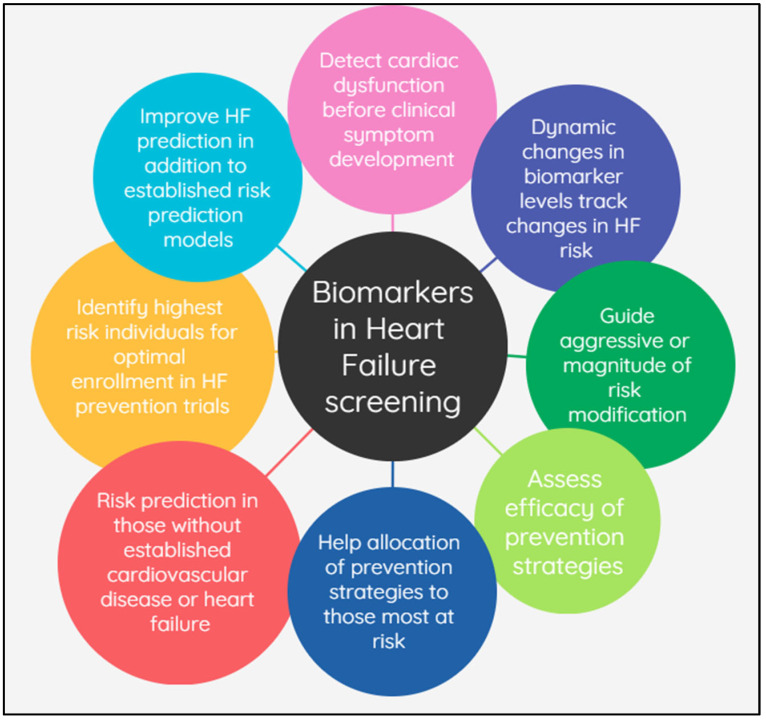
The various roles of biomarkers in heart failure prediction and prevention.

Several biomarkers have been identified as markers of cardiac dysfunction or injury and are useful for HF risk prediction and screening [7]. Studies also demonstrated that biomarkers can improve prognostication and risk prediction [8,9], and repeated measurements and trends of biomarkers over time can reflect dynamic changes in HF risk [10,11,12]. Biomarkers have also been studied to guide the intensity of risk factor management and assess the efficacy of prevention strategies [13,14]. Various roles and contributions of biomarkers in HF prediction and prevention are summarized in Figure 1. A few key studies that demonstrate their potential utility are discussed below. 

### 2.1. Brain Natriuretic Peptide (BNP) and N-Terminal-Pro Hormone BNP (NT-proBNP)

#### 2.1.1. HF Prediction and Prevention 

BNP and NT-proBNP are released from the myocardium during end-diastolic wall stress. They are both breakdown products of the prohormone pre-proBNP. Their downstream effects include vasodilation, natriuresis, and diuresis. In the next few paragraphs, we will discuss some of the studies that have demonstrated the utility of these biomarkers in HF prediction. 

In a prospective study of 3346 patients without HF, a mean follow-up of 5.2 years, BNP > 20 pg/mL in men, and BNP > 23.3 pg/mL in women was found to be associated with a significantly higher risk of new-onset HF (HR 3.07, *p* = 0.002) [7]. Each increment of 1 SD in log BNP levels was associated with a 77% increase in the risk of heart failure (*p* < 0.001). 

In an analysis of the Atherosclerosis Risk in Communities (ARIC) cohort, the addition of NT-proBNP significantly improved the Framingham Heart Study (FHS), Health Aging and Body Composition (ABC), and ARIC HF risk prediction models, with the categorical Net Reclassification Index (NRI) being 0.18, 0.12, and 0.13, respectively [8]. Similarly, the addition of NT-proBNP improved HF risk prediction with a categorical NRI = 0.37 in the Multi-Ethnic Study of Atherosclerosis (MESA) cohort study [9]. The improvement was primarily due to the upward reclassification of individuals who subsequently developed HF.

#### 2.1.2. Dynamic Changes in Biomarker Levels Reflect Changes in HF Risk

NT-proBNP levels frequently change over time, and these were found to reflect dynamic changes in HF risk [10]. Using the ARIC cohort, Jia et al. used NT-proBNP levels, measured 6 years apart and reported that the percent change in NT-proBNP, per 1-SD increase, was positively associated with an increased risk of HF (HR 1.06, 95% CI 1.02–1.10) and death (HR 1.05, 95% CI 1.03–1.08). Individuals with more than a 25% increase in NT-proBNP between the two visits had a higher risk of HF hospitalization and death compared with those with a 25% or smaller change in biomarkers. 

Similarly, in the Heart and Soul study, in participants with stable coronary artery disease, participants with 5-year changes in the highest quartile (≥223 pg/mL increase in NT-proBNP) had an almost 4-fold greater risk of subsequent HF or CV death than those in the lowest quartile of ≤−5 pg/mL (HR 3.8; 95% CI 2.0–7.3; *p* < 0.001) [11]. A stable NT-proBNP level predicted a low risk of subsequent events.

Additionally, in an analysis from the Cardiovascular Health Study (CHS), a significant increase in NT-proBNP (>25% increase) at follow-up (2–3 years) was associated with an increased risk of HF (HR 2.06; 95% CI 1.56–2.72) and CVD (HR 1.88; 95% CI: 1.37–2.57), while those with a significant decrease in NT-proBNP (>25% decrease to < or = 190 pg/mL) had a lower risk of HF (HR 0.58; 95% CI: 0.36–0.93) and CVD (HR 0.57; 95% CI 0.32–1.01) compared with individuals with unchanged values [12]. 

These findings underline the potential role of serial measurements of biomarkers to characterize changes in risk over time. Those with persistently elevated or rising biomarkers could represent those with a higher risk and are thus targets for more aggressive interventions compared to those with significant reductions in biomarkers who may have a lower risk and may be indicative of effective risk factor modification. 

In summary, there is robust evidence across several studies that support the role of natriuretic peptides in identifying individuals at high risk of incident HF, thereby expanding their role from diagnosis to predicting risk.

### 2.2. Cardiac Troponin

Cardiac troponins (cTns) play an integral role in regulating myocardial contraction, including troponin C (cTnC) in calcium binding, troponin I (cTnI) in inhibition, and troponin T (cTnT) in tropomyosin binding. Although cTns came to prominence for their value in diagnosing myocardial infarction, they can be released in patients with HF secondary to subendocardial ischemia, myocardial injury, or cardiac wall stress. 

In a study including 9698 ARIC study participants aged 54–74 years who were free from coronary heart disease (CHD), stroke, and HF, Saunders et al. found that compared with participants with undetectable levels, those with high-sensitive cardiac troponin T (hs-cTnT) levels in the highest category (≥0.014 μg/L) had a significantly increased risk of HF (HR = 5.95; 95% CI 4.47–7.92) after adjusting for traditional cardiac risk factors, kidney function, high-sensitivity C-reactive protein (CRP), and NT-proBNP [15].

Similarly, in an analysis of the CHS cohort including 4221 adults aged 65 years or older without prior HF with a median follow-up of 11.8 years, DeFilippi et al. found that among participants with the highest cTnT concentrations (>12.94 pg/mL), there was a higher risk of HF (adjusted hazard ratio (aHR), 2.48; 95% CI, 2.04–3.00) compared to participants with undetectable cTnT levels [16]. In terms of dynamic changes, a rising trajectory of cTnT was associated with a greater risk of HF, while a declining trajectory was associated with a lower risk of HF. Individuals with a subsequent increase in cTnT of more than 50% were associated with a greater risk of HF (aHR, 1.61; 95% CI, 1.32–1.97), and a decrease of more than 50% was associated with a lower risk of HF (aHR, 0.73; 95% CI, 0.54–0.97) compared with participants with 50% or less change.

Nambi et al. compared several HF risk prediction models and found that adding cTnT and NT-proBNP to the ARIC HF model significantly improved HF prediction. The area under the curve (AUC) increased by 0.040 and 0.057; 38% of men and 32% of women were reclassified, with continuous NRIs of 50.7% and 54.7% in women and men, respectively [17]. They also found that a simplified HF prediction model, termed the ‘laboratory model’ (cTnT, NT-proBNP, age, and race), was comparable to the ARIC HF model with no statistically significant differences in the AUC, NRI, or integrated discrimination improvement (IDI). Overall, they concluded that a simplified model using just the biomarkers, age, and race, which can be easily implemented, can help identify individuals at a high risk of HF, although the best approach would be factoring in both clinical variables and the biomarkers. 

### 2.3. Albuminuria 

Albuminuria, the presence of albumin in urine, reflects structural damage in the glomeruli and has been identified as an early marker of glomerular damage before a decline in the glomerular filtration rate (GFR) occurs [18]. A progressive increase in albuminuria has been associated with an increased risk of HF [18]. The pathophysiology between albuminuria and HF is multifactorial and stems from the combination of endothelial damage, tubular damage, and comorbid conditions (e.g., hypertension, obesity, diabetes mellitus) that ultimately result in an inflammatory state and volume overload due to activation of the renin–angiotensin–aldosterone system. The Health ABC score, Framingham risk score, and Cardiovascular Health Score are a few HF risk prediction scores that already incorporate albuminuria.

In a post hoc analysis of the RENAAL (Reduction of Endpoints in NIDDM with the Angiotensin II Antagonist Losartan) trial, including 1513 patients with type 2 diabetes and nephropathy with a mean follow-up time of 3.4 years, patients with high baseline albuminuria (≥3 g/g creatinine) levels had a 2.7-fold increased risk of incident HF compared with patients with low albuminuria [18]. Similar results, demonstrating an association between albuminuria and an increased risk of incident HF, were also reported in the FHS (HR 1.71, 95% CI: 1.56–4.78) and MESA (HR 2.73, 95% CI: 1.56–4.78) [18]. 

In a study of ARIC participants, Blecker et al. assessed the association between albuminuria, which they defined as optimal (<5 mg/g), intermediate normal (5–9 mg/g), high normal (10–29 mg/g), microalbuminuria (30–299 mg/g), macroalbuminuria (≥300 mg/g), and risk of heart failure. The authors noted that increasing levels of albuminuria were associated with a graded increased risk of heart failure categorized as intermediate normal (aHR, 1.54; 95% CI, 1.12–2.11), high normal (aHR, 1.91; 95% CI, 1.38–2.66), microalbuminuria (aHR, 2.49; 95% CI, 1.77–3.50), and macroalbuminuria (aHR, 3.47; 95% CI, 2.10–5.72) [19]. 

These studies support that albuminuria or the urinary albumin to creatinine ratio (UACR) represents an effective strategy for predicting HF risk [18].

### 2.4. Glomerular Filtration Rate: Cystatin C and Creatinine 

Renal function markers with potential utility for HF risk prediction include cystatin C and creatinine. In a study of individuals without HF enrolled in the United Kingdom (UK) Biobank cohort, Nowak et al. reported that the addition of the cystatin C-based estimated glomerular filtration rate (cysC-eGFR) and the urine albumin-to-creatinine ratio to the ARIC study HF risk score led to a significant improvement in the risk discrimination ratio (ΔC = 0.019 (95% CI, 0.015–0.022)) [20]. Similarly, in an analysis of CHS with 5888 elderly people >65 years old (average 73 ± 5) and an average follow-up of 5.5 years (median 6.3), Gottdiener et al. reported that a serum creatinine > 1.4 mg/dL had a relative risk of 1.5 (95% CI 1.17–1.92, *p* = 0.001) in the prediction of HF [21]. 

### 2.5. Other Exploratory Biomarkers (e.g., Inflammatory and Oxidative Markers: CRP, Galectin-3, Soluble ST2 (sST2), and Growth Differentiation Factor (GDF-15))

Several other biomarkers associated with inflammatory and oxidative states, such as sST2, GDF-15, galectin-3, and CRP, are predictive of HF development. sST2 is associated with myocardial fibrosis and adverse remodeling [22]. GDF-15 is a member of the TGF-β family and is released in response to oxidative stress, proinflammatory cytokines, ischemia, or mechanical stretch. The increased expression of galectin-3 is implicated in pathological remodeling and inflammatory processes, including neutrophil adhesion, monocyte chemoattraction, and mast cell activation [23]. 

Increased sST2 levels have been associated with greater LV dimension, poorer LV and RV function, hemodynamic decompensation, and increased rates of mortality [24,25]. In the Framingham Offspring Cohort, increasing levels of galectin-3 were associated with an increased risk of incident HF (HR 1.28 per 1 SD increase in log galectin-3; 95% CI 1.14–1.43; *p* < 0.0001), which remained significant after adjustment for clinical variables and BNP (HR 1.23; 95% CI 1.04–1.47; *p* = 0.02) [26]. In another population-based cohort, galectin-3 was associated with all-cause mortality (HR: 1.12, *p* < 0.001), cardiac death (HR: 1.15, *p* = 0.033), and heart failure (HR: 1.10, *p* = 0.049) [27]. 

Among 3428 subjects in the FHS, elevated concentrations of sST2, hs-TnI, GDF-15, and BNP were independently associated with incident HF during a mean follow-up of 11 years [28]. 

Regarding CRP, in a study of four longitudinal community-based cohorts, including the Cardiovascular Health Study, the Framingham Heart Study, the Multi-Ethnic Study of Atherosclerosis, and the Prevention of Renal and Vascular End-stage Disease study, de Boer et evaluated the associations of twelve cardiovascular markers with incident HF. They identified CRP to be associated with incident heart failure with reduced ejection fraction development (HR, 1.19; 95% CI, 1.11–1.28; *p* < 0.001) [29]. Similarly, in the ABC study (Health, Aging, and Body Composition), CRP was found to be associated with new-onset HF [30]. 

## 3. Biomarkers in HFpEF vs. HFrEF

HF is a heterogeneous syndrome, with different risk factors and pathophysiological processes contributing to different phenotypes of the heart failure spectrum that range from heart failure with preserved ejection fraction (HFpEF) to heart failure with reduced ejection fraction (HFrEF). Different biomarkers have been associated with the development of incident HFpEF versus HFrEF. 

In a study of four community-based cohorts, including CHS, FHS, MESA, and the Prevention of Renal and Vascular End-stage Disease (PREVEND) studies, De Boer et al. investigated the association of twelve biomarkers with HFrEF and HFpEF. Biomarkers predictive of HFrEF versus HFpEF onset were quite different; hs-cTn had a stronger association with incident HFrEF (HR: 1.37) than HFpEF (HR: 1.11) [29].

In the St Vincent’s Screening to Prevent Heart Failure (STOP-HF) study, Watson et al. found that a combination of biomarkers, high-sensitivity troponin I (hsTnI), BNP, and galectin-3 significantly predicted future HFpEF using both baseline (AUC 0.82 (0.73, 0.92)) and follow-up data (AUC 0.86 (0.79, 0.94)). The authors reported a novel relative risk model for new-onset HFpEF development stratified by quintiles of BNP, median high sensitivity troponin I (hsTnI), and galectin-3 thresholds [31]. Brouwers et al. evaluated the prognostic value of 13 biomarkers to predict HFrEF and HFpEF in 8569 HF-free participants and found that except for a modest effect of cystatin-C, none of the other biomarkers were associated with an increased risk of HFpEF [32]. 

As our understanding of HF development evolves, different biomarkers associated with specific risks for different HF phenotypes and etiologies may be identified. 

## 4. Race and Biomarkers

The importance of race-specific models is being recognized for HF risk prediction [33]. 

Segar et al. analyzed four large cohort studies, ARIC, DHS, Jackson Heart Study (JHS), and MESA, and investigated the effect of race on various HF risk prediction models [33]. Among several risk factors, natriuretic peptide levels were the most important predictor of HF risk across races, followed by troponin, glycemic parameters, and socioeconomic factors in Black adults. The ECG-based measure of voltage and left ventricular hypertrophy (LVH) and classical cardiovascular risk factors (CVRFs) were stronger predictors of HF risk in White adults. 

## 5. Multi-Biomarker and Multimodality Approach 

With HF being a heterogeneous clinical syndrome, a multi-biomarker and multimodality approach is recognized as an appealing strategy to incorporate different pathological pathways and improve risk prediction [14,28,34,35]. 

In the Biomarkers for Cardiovascular Risk Assessment in Europe consortium study, the addition of hs-cTnI and NT-proBNP to a prognostic model consisting of CVRFs improved HF prediction, with the best predictive value for incident HF (C-index = 0.862) being achieved by combining CVRFs with both hs-cTnI and NT-proBNP [36]. Similarly, in an analysis of the CHS cohort, the addition of NT-proBNP and echocardiographic features to the clinical Health ABC HF risk score led to a 16.3% NRI preduction of 5-year HF risk [35]. Additionally, in the FHS, a ‘multimarker’ score composed of sST2, troponin, GDF-15, CRP, and BNP was found to be associated with incident HF. Individuals with multimarker scores in the highest quartile had a 6-fold risk of heart failure (6.2; 95% CI, 2.6–14.8; *p* < 0.001) [28]. The addition of the multimarker score to clinical variables led to significant increases in the c-statistic (*p* = 0.005 or lower) and NRI (*p* = 0.001 or lower). 

In an analysis of the ARIC cohort data according to the new 2022 AHA/ACC/HFSA guidelines, Jia et al. demonstrated that the combination of NT-proBNP, hsTnT, and echocardiography identified individuals at the highest HF risk and death [34]. Using NT-proBNP (<125 pg/mL versus ≥125 pg/mL), high-sensitivity troponin T (<14 ng/L versus ≥14 ng/L), and abnormal cardiac structure/function by echocardiography, individuals were classified as Stage A_new_ and Stage B_new_ HF. Stage B_new_ was further sub-classified into elevated biomarkers only (Stage B_biomarkers only_), abnormal echocardiogram only (Stage B_echo only_), and abnormalities in both (Stage B_echo+biomaker_). Elevated biomarker levels led to 21.1% of study participants being reclassified from Stage A to Stage B_new_ HF. Compared with Stage A_new_, patients in Stage B_new_ were associated with an increased risk of incident HF (HR:3.70, 95% CI 2.58–5.30) and death (HR 1.94, 95% CI 1.53–2.46). Stage B_biomarkers only_ and Stage B_echo only_ were both associated with increased HF risk, but only Stage B_biomarkers only_ was associated with increased death. Stage B_echo+biomaker_ was the most predictive of HF (HR 6.34, 95% CI 4.37–9.19) and death (HR 2.53, 95% CI: 1.98–3.23). 

In an analysis of the ARIC, MESA, and DHS cohorts, Pandey et al. reported the utility of a multi-biomarker and multimodality score to guide the allocation of heart failure prevention therapies [14]. Using a biomarker score composed of hs-cTnT ≥6 ng/L, NT-pro-BNP ≥125 pg/mL, high-sensitivity CRP ≥3 mg/L, and LVH by electrocardiography (with 1 point for each abnormal parameter), the authors reported that the 5-year risk of HF increased in a graded fashion with an increasing biomarker score, with the highest risk being among those with scores of ≥3 (5-year risk of HF in patients with diabetes: 12.0%; patients with pre-diabetes: 7.8%). Based on a theoretical analysis, they found that in those with a greater biomarker score, the numbers-needed-to-treat to prevent HF using SGLT-2i was lower, highlighting the opportunity for more targeted allocation of heart failure prevention therapies through better risk stratification. 

Some studies report on the utility of LVH, diagnosed by ECG or MRI, in combination with biomarkers to predict HF [37]. In a study of Dallas Heart Study participants, the authors demonstrated that left ventricular hypertrophy (LVH) diagnosed by magnetic resonance imaging combined with either elevated cTnT (≥3 pg/mL) or NT-proBNP (75th age- and sex-specific percentile) predicted HF and CV death [37]. The incidence of HF or death among patients with LVH and elevated cTnT over 8 years was 21% compared to 6% in those with LVH and negative cTnT (*p* < 0.0001). In patients with LVH and elevated NT-proBNP, the incidence was 20% compared to 7% in patients with LVH and negative NT-proBNP. Individuals who had LVH and either cTnT or NTproBNP elevation were at a 4-fold higher risk of HF or CV death after multivariable adjustment for CV risk factors, renal function, and LV mass compared with those who did not have LVH and elevated biomarkers. In a pooled analysis of the ARIC, DHS, and MESA studies, the role of elevated cardiac biomarkers, hs-cTnT ≥ 6 ng/L and NT-proBNP ≥ 100 pg/mL, and ECG LVH (defined as malignant LVH) to predict incident HF was investigated [38]. Compared with participants without LVH, in those with malignant LVH, aHR for HF was 2.8 (95% CI, 2.1–3.5), and in those with LVH and normal biomarkers, it was 0.9 (95% CI, 0.6–1.5). 

The combination of risk prediction scores, biomarkers, echocardiographic, and electrocardiographic features enhances the risk prediction for heart failure and can be used to identify the highest-risk individuals. This would help identify strategies to test HF prevention for those who are at the highest risk and target these therapies to those who are most likely to benefit from them. 

## 6. Strategies to Prevent HF

Below, we highlight a few studies that demonstrate the possibility of preventing or altering heart failure disease course. Studies have shown that effective risk factor management, including optimal hypertension management, diabetes management, and weight loss in obesity, can reduce the incidence of HF [39,40,41]. 

In the St Vincent’s Screening to Prevent Heart Failure (STOP-HF) study [39], 1374 participants with significant cardiovascular comorbidities were randomly assigned to standard care (n = 677) or intervention with BNP screening (n = 697). In the intervention group, those with a BNP ≥50 ng/L underwent echocardiography and referral to cardiology. After a mean follow-up of 4.2 years, the intervention group received more renin–angiotensin–aldosterone system (RAAS) therapy (56.5% vs. 49.6%; *p* = 0.01) and overall was associated with a lower incidence of asymptomatic LV dysfunction with or without new HF diagnosis (5.3% vs. 8.7%, OR 0.55, 95% CI 0.37–0.82; *p* = 0.003).

In patients at a high risk of future HF development, including those with type 2 diabetes and chronic kidney disease, sodium–glucose co-transporter 2 inhibitors (SGLT2is) have been found to lower the risk of incident HF [42,43]. In a propensity-matched cohort study of patients with diabetes, the risk of incident HF was significantly lower in the SGLT2i cohort compared to the non-SGLT2i cohort (HR 0.70, 95% CI 0.68–0.73) [43]. 

In the FIGARO-DKD (Finerenone in Reducing Cardiovascular Mortality and Morbidity in Diabetic Kidney Disease) trial, a study of 7352 patients with type 2 diabetes, albuminuric chronic kidney disease and no symptomatic heart failure at baseline in the finerenone versus placebo group was associated with a reduced risk of new-onset HF (1.9% versus 2.8%; HR, 0.68 (95% CI, 0.50–0.93); *p* = 0.0162) [44]. There was a 29% lower risk of first hospitalization for HF in the finerenone versus placebo group (HR, 0.71 (95% CI, 0.56–0.90); *p* = 0.0043). 

In another study, healthy behaviors, as defined by AHA’s Life’s Simple 7 (LS7) metrics, were associated with a reduced risk of HF development [45,46]. In a study by the EPIC-NL (European Prospective Investigation Into Cancer and Nutrition-Netherlands) cohort, including 37,803 participants (mean age: 49.4 ± 11.9 years, 74.7% women), LS7 scores were calculated by assigning 0, 1, or 2 points for smoking, physical activity, body mass index, diet, blood pressure, total cholesterol, and blood glucose [46]. An overall ideal score (11 to 14 points) was present in 23.2% of participants, an intermediate score (9 or 10 points) in 35.3%, and an inadequate score (0 to 8 points) in 41.5%. Over a median follow-up period of 15.2 years, ideal (HR 0.45, 95%CI: 0.34 to 0.60) and intermediate (HR 0.53, 95% CI 0.44 to 0.64) LS7 scores were associated with a reduced risk of HF compared with the inadequate category. Having an ideal LS7 score was associated with a 55% lower risk of HF compared with an inadequate LS7 score. 

There are also emerging data that individuals with elevated biomarkers may derive a higher benefit from risk factor modification than those without elevated biomarkers in HF prevention. In a post hoc analysis of the Systolic Blood Pressure Intervention Trial (SPRINT), intensive systolic blood pressure (SBP) control led to greater absolute risk reduction (ARR) in death and HF in the sub-group with elevated hscTnT (≥14 ng/L) and NTproBNP (≥125 pg/mL) compared to those with neither biomarker elevation: 7.8% (95% CI 3.3–11.3%) vs. 1.7% (95% CI 0.8–2.3%), respectively [13]. In another analysis of the SPRINT study, malignant LVH (ECG with LVH, hscTnT ≥ 14 ng/L and NTproBNP ≥ 125 pg/mL) was associated with an increased risk of ARR of HF and death (4.4%) compared to 1.2% in those without elevated biomarkers or LVH. Intensive SBP lowering also reduced the incidence of malignant LVH over 2 years (2.5% vs. 1.1%; OR: 0.44; 95% CI: 0.30–0.63) [47]. 

## 7. Directions for Future Research 

Many questions remain unanswered regarding biomarkers and their role in heart failure prediction and screening. The cost-effectiveness and risks of HF screening and their impact on quality of life and mortality are not well studied and warrant further research. A better understanding of biomarker profiles in different HF subtypes and patient populations, as well as the timing, specific combinations, and frequency of biomarker measurements, is also required. Further research to better characterize HF pathophysiology, etiology, and phenotypes can also identify further specific preventive strategies. 

## 8. Conclusions

The prevalence and burden of HF are rapidly increasing, and HF prediction and prevention have become more important than ever. The 2022 ACC/AHA/HFSA Guidelines provide recommendations for BNP or NT-proBNP-based screening, followed by team-based care to optimize GDMT, as a strategy to prevent the development of new-onset HF. Large studies with biomarkers, especially natriuretic peptides, cardiac troponin, and albuminuria, support the utility and efficacy of these biomarkers for HF risk prediction and prevention, Further research will help better define the role of additional inflammatory, oxidative, renal, and other markers. 

There are studies suggesting that biomarker profiles associated with incident HFpEF and HFrEF might be different. The importance of differences in biomarker risk profiles based on race is increasingly recognized. Further research is required to better characterize biomarkers associated with different risk factors and pathways contributing to HF development across age, sex, and race. 

A multi-biomarker and multimodality approach, incorporating multiple biomarkers added to risk prediction scores and clinical information (such as electrocardiograms, MRIs, and echocardiograms), are evolving as important strategies to enhance risk prediction. 

Studies have demonstrated the possibility of preventing or altering heart failure disease course with interventions such as treatment with GDMT, healthy behaviors, as defined by AHA’s Life’s Simple 7, and optimal comorbidity management, including intensive blood pressure management. Individuals with elevated biomarkers have been shown to derive the most benefit from risk factor modification in HF prevention. Thus, biomarkers help identify individuals at the greatest risk of developing HF and support the more targeted implementation of preventive strategies. As new data emerge, the utility of biomarkers in HF screening and prevention likely will continue to evolve and help reduce the burden of heart failure.
